# Dynamic gesture recognition based on 2D convolutional neural network and feature fusion

**DOI:** 10.1038/s41598-022-08133-z

**Published:** 2022-03-14

**Authors:** Jimin Yu, Maowei Qin, Shangbo Zhou

**Affiliations:** 1 College of Automation, Chongqing University of Posts and Telecommunications, Chongqing, 400065 China; 2 College of Computer Science, Chongqing University, Chongqing, 400044 China

**Keywords:** Mathematics and computing, Computer science

## Abstract

Gesture recognition is one of the most popular techniques in the field of computer vision today. In recent years, many algorithms for gesture recognition have been proposed, but most of them do not have a good balance between recognition efficiency and accuracy. Therefore, proposing a dynamic gesture recognition algorithm that balances efficiency and accuracy is still a meaningful work. Currently, most of the commonly used dynamic gesture recognition algorithms are based on 3D convolutional neural networks. Although 3D convolutional neural networks consider both spatial and temporal features, the networks are too complex, which is the main reason for the low efficiency of the algorithms. To improve this problem, we propose a recognition method based on a strategy combining 2D convolutional neural networks with feature fusion. The original keyframes and optical flow keyframes are used to represent spatial and temporal features respectively, which are then sent to the 2D convolutional neural network for feature fusion and final recognition. To ensure the quality of the extracted optical flow graph without increasing the complexity of the network, we use the fractional-order method to extract the optical flow graph, creatively combine fractional calculus and deep learning. Finally, we use Cambridge Hand Gesture dataset and Northwestern University Hand Gesture dataset to verify the effectiveness of our algorithm. The experimental results show that our algorithm has a high accuracy while ensuring low network complexity.

## Introduction

In addition to the use of language, human beings use gestures as an indispensable communication tool when communicating and conveying information. In the field of computer vision, gesture recognition is also one of the most important and topical problems and has been used in many fields, such as human–computer interaction^1^, virtual reality systems^2,3^, and sign language recognition^4^. Traditional gesture recognition requires the use of data gloves^5^ or other relevant external devices to collect the spatial position changes of the hand and arm joints to determine the real intention of the wearer. This traditional approach has high recognition accuracy, but is costly and poorly scalable and easy to use. In recent years, with the rapid development of computer vision, techniques to obtain accurate gesture recognition without the aid of external devices have been proposed one after another. Among the many gesture recognition methods, they can be divided into two categories: static gesture recognition and dynamic gesture recognition. Static gesture recognition methods have significant limitations^6,7^. It can only simply recognize a single shape of the hand, but cannot obtain its spatial and state variation. For example, it can recognize whether the hand is in a ‘held’ or ‘unfolded’ state, but not the process of change from ‘held’ to ‘unfolded’. Dynamic gesture recognition considers the spatial and temporal information of the whole process and can recognize the change process of the target object, which has important research implications.

Before the rapid development of deep learning, research on dynamic gesture recognition mainly relied on manual extraction of features and then building sequence models for recognition. The accuracy of this approach was low and inefficient, so the commonly used methods now rely mainly on deep learning. As dynamic gesture data is generally presented in video form, it is difficult for 2D convolutional neural networks (CNNs) to extract sufficient feature information, which has prompted researchers to explore new directions. With Du Tran et al.^8^ proposing the C3D network model, which solved the problem of retaining both temporal and spatial features, the dynamic gesture recognition problem was widely solved by deep learning methods. the C3D model was also the earliest 3D CNN model. However, the 3D CNN model is too complex, the training time is too long and the hardware requirements are very high. These key issues still constrain the development of dynamic gesture recognition.

To improve the problem of large network model parameters and training difficulties, we propose a strategy based on dual-channel 2D CNN and feature fusion. First, the optical flow frames of the video data were extracted using the fractional Horn and Schunck (HS) optical flow method^9^, and then five original key frames and optical flow key frames were extracted separately using an improved clustering algorithm and subjected to a horizontal stitching operation. Finally, the stitched original keyframe feature map is used to represent the spatial features in the video data, and the optical flow keyframe stitching map represents the temporal features in the video data. This method not only preserves the spatial and temporal features of the video, but it also greatly reduces the size of the dataset and improves the training efficiency. Most current algorithms on dynamic gesture recognition using 2D CNN serialize the video datasets as a chart or a single image, which loses the information on the variation of key spatio-temporal features. Our proposed algorithm intuitively extracts the temporal and spatial information in the video datasets and fuses the two, making full use of the key features in the video datasets. Experimental results show that our proposed strategy is accurate and effective on the Cambridge Hand Gesture dataset^10^ and Northwestern University Hand Gesture dataset^11^. To summarize, the main contributions of this paper are:

- An improved the HS model is proposed with the fractional order method, in which fractional-order and deep learning are creatively combined;
- An improved clustering algorithm is proposed based on a tradition model, which can effectively extract the keyframes of complex actions;
- A strategy for network input is proposed to use the original keyframe mosaic image and the optical flow keyframe mosaic image instead of the video data, which effectively reduces the size of the data set and the difficulty of training.

## Related work

One of the most popular technologies, gesture recognition has been in development for decades. During this time, gesture recognition has developed to an unprecedented level and various novel algorithms have been proposed. Here we present a relevant summary in two parts: algorithms that do not use deep learning and algorithms that do.

### Gesture recognition without deep learning

Wang et al.^12^ used the Hidden Markov Model algorithm for modeling and reconstructing the dynamic gesture trajectories. The global feature is represented by an invariant curve moment, and the local feature is represented by a direction to represent the gesture trajectory for recognition. Oreifej et al.^13^ used the histogram method to replace the sequence model to represent the space and time information represented in the depth sequence to achieve the purpose of identification. Chen et al.^14^ used the hand segmentation algorithm to obtain the shape feature and time feature of the data set, used the Fourier descriptor method to extract the feature vector from it and used the hidden Markov chain for recognition. Rahman et al.^15^ used biorthogonal wavelet transform to preprocess the image and finally constructed a multi-class support vector machine for recognition. These forementioned methods already have a certain degree of accuracy, but the robustness is poor.

### Gesture recognition with deep learning

Cheng et al.^16^ combined sEMG feature images and convolutional neural networks for gesture recognition, which effectively addressed the limitations of traditional machine learning in sEMG gesture recognition and combined with 1-dim convolutional kernel to extract deep abstract features to improve the recognition effect. Liao et al.^17^ analyzed the single multi-box detector (SSD) algorithm and compared the front-end networks. MobileNets was chosen as the front-end network and the MobileNets-SSD network was improved. Effectively improves the problem of hand shading. Li et al.^18^ extracted the sEMG signals of forearm muscles based on human hand movements and used the root mean square, wavelength and nonlinear feature sample entropy in the time domain as the three feature values. Finally, high accuracy rate of hand motion recognition was successfully achieved by GRNN and SVM. Huang et al.^19^ improved the YOLO v3 algorithm to determine whether a worker meets the criteria for wearing a helmet based on an empirical threshold. There was a more significant improvement compared with the original YOLO v3 algorithm. huang et al.^20^ designed a framework for semantic segmentation network of images with joint target detection. By adding parallel operations of semantic segmentation branches to the target detection network, a multi-vision task combining object classification, detection and semantic segmentation is innovatively implemented. It effectively improves vision tasks in complex environments. Yang et al.^21^ proposed a multistream residual network (MResLSTM) for dynamic hand action recognition. The network combines residual and convolutional short-term memory models into a unified framework and uses a strategy of clockwise grouped convolution and channel shuffling to reduce the number of network computations. The final result is a highly accurate recognition. Weng et al.^22^ developed a cascaded two-level convolutional neural network model and proposed an Angle-Net model to finely estimate the grasping angle in response to the lack of accuracy of previous methods in pose detection. It effectively improves the problem of multiple objects stacked and obscured by each other, which makes it difficult for the robot to recognize the target when grasping. Duan et al.^23^ constructed a weighted adaptive algorithm incorporating different features to optimize the RGB-D information processing. Finally, the feasibility and robustness of the algorithm are verified by means of experiments. Liu et al.^24^ proposed a new end-to-end dual-stream structure called the fusion of space-time network. This network closely fuses spatial and temporal features to obtain rich spatio-temporal information and achieve accurate recognition results. Karpathy et al.^25^ proposed a multi-resolution CNN network that can be used to process large-scale data. Compared with the network using strong features, its performance has been significantly improved. Simonyan et al.^26^ constructed a dual-stream CNN model. The two-channel model is a spatial network trained on the original frame and a temporal network trained on the optical flow frame. Inspired by the dual-stream convolutional network, Wang et al.^27^ constructed a temporal segment network (TSN), which is a new video-based action recognition framework, which aims to adopt a segment-based sampling and aggregation module Model the long-distance time structure. Molchanov et al.^28^ combined a high-resolution network (HRN) and low-resolution network (LRN) to construct a new CNN-based classification network. The recognition result is obtained by the probabilistic fusion of the two branches. Gesture recognition with deep learning has great advantages in terms of stability and scalability and is the mainstream method in the field of computer vision.

## Proposed approach

### Statement

Confirming that all experiments were performed in accordance with relevant guidelines and regulations.

### Network structure

**Figure 1:**
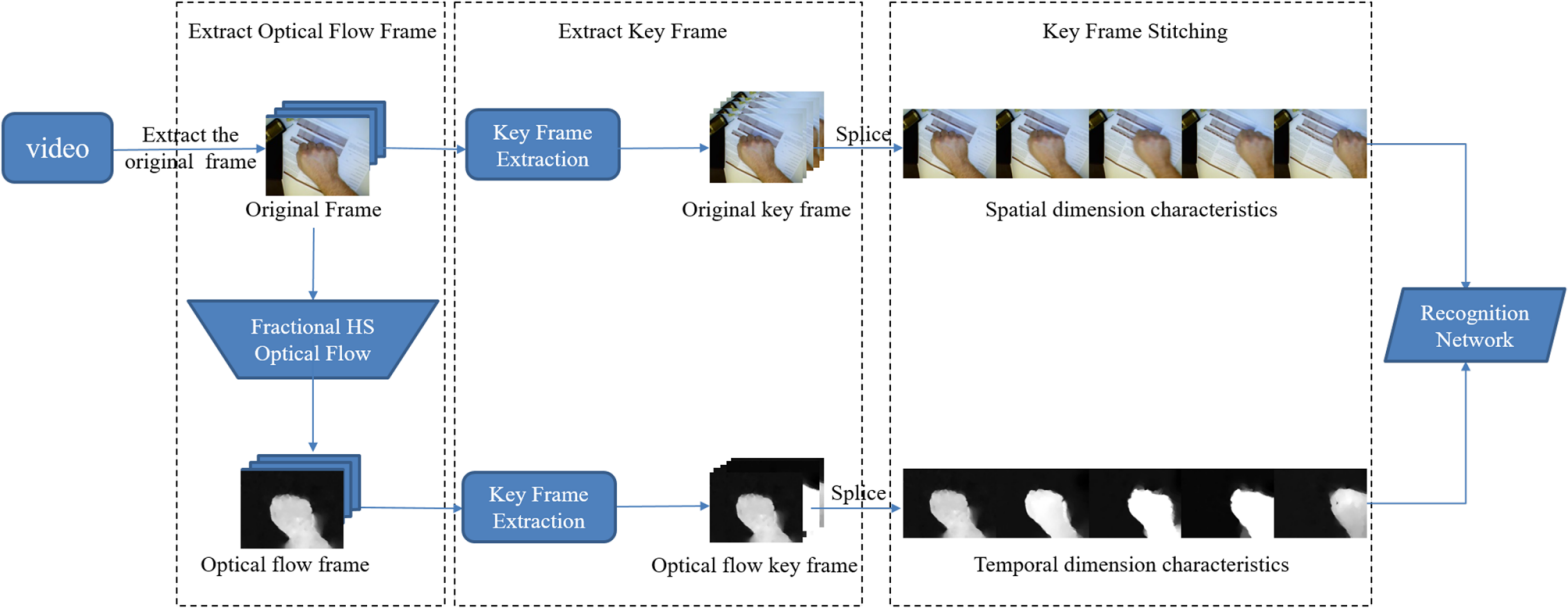
Overview of the proposed feature fusion strategy.

When performing dynamic gesture recognition, in order to enable 2D CNN to analyze the spatial and temporal information of video data at the same time, we propose a fusion strategy as shown in Fig. 1. Firstly, we extract the original frames of the video. Then the fractional HS optical flow method is used to extract the optical flow frames corresponding to the original frame. Finally, the proposed clustering algorithm is used to extract original frames and optical flow frames as the keyframes of the video and carry out the horizontal splicing operation. For a video data, its spatial dimension feature will be represented by the spliced original keyframe image, and time dimension feature be represented by the optical flow keyframe image. We use the feature fusion of the two kind keyframe images to represent the feature of the video data, and send it to the 2D recognition network for recognition.

**Figure 2:**
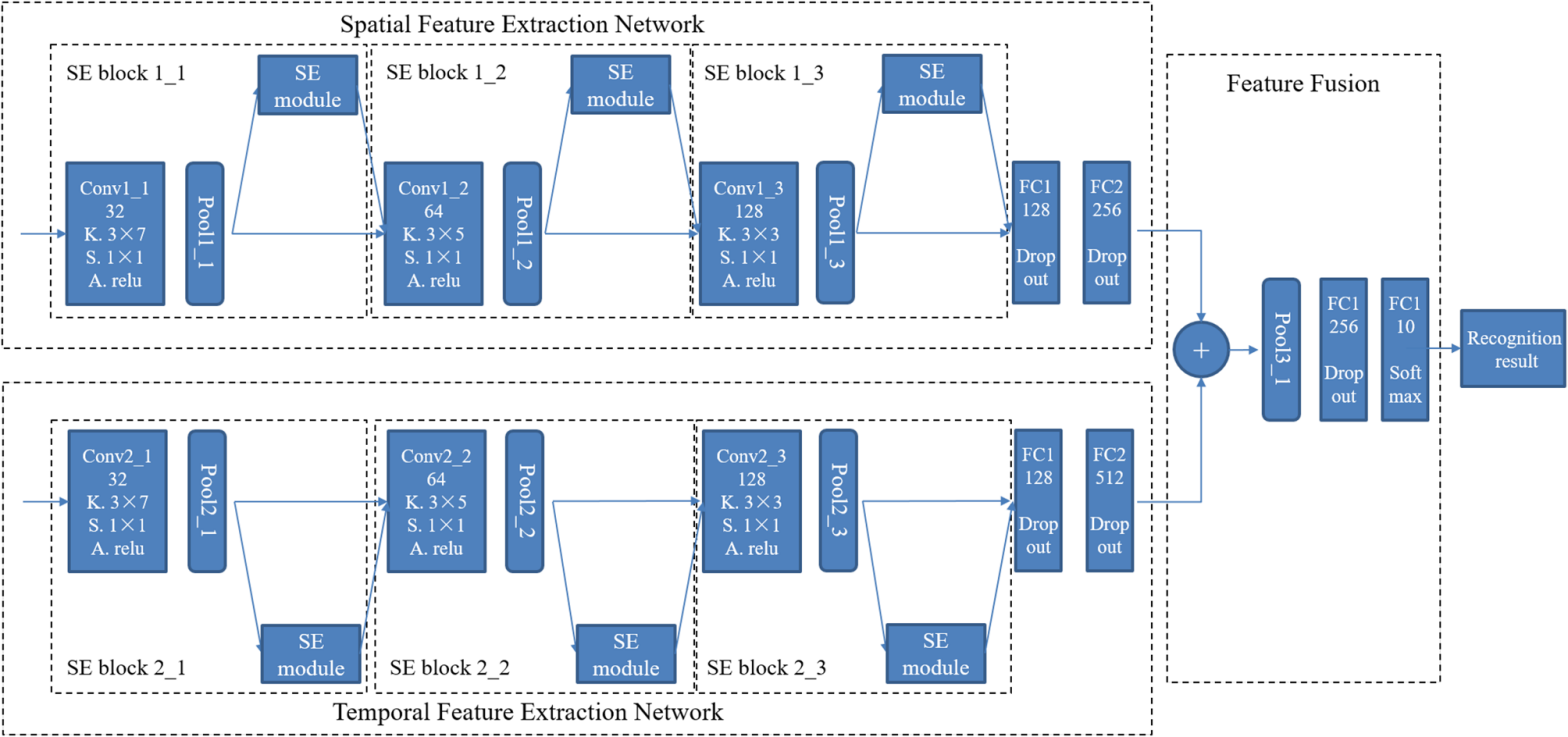
Recognition network structure.

The structure of the recognition network is shown as in Fig. 2. The spatial feature extraction network and the temporal feature extraction network have the same structure, and both are composed of three Squeeze-and-Excitation (SE) blocks. Since we have done a horizontal splicing operation on the keyframes, the length of the input picture is much greater than the width, so the convolutional layer of SE block 1_1 consists of 32 $3\times 7$ convolution kernels with a step size of 1. Enable the convolutional layer to extract more features in the lateral direction. To enable the extracted features to better, reflect the global information of the feature map, we have added the SE module to allow the network to perform feature recalibration. Through the SE module, the network can selectively emphasize useful global features and suppress less useful features. The convolutional layer of SE block 1_2 is composed of 64 $3\times 5$ convolution kernels with a step length of 1. The convolutional layer of SE block 1_3 consists of 128 $3\times 3$ convolution kernels with a step size of 1. Finally, after two full connections, spatio-temporal feature fusion is performed and the fused features are input to the full connection layer to realize the classification of gesture actions. To reduce the possibility of network overfitting, a batch normalization layer and a dropout layer are added to the network.

**Figure 3:**
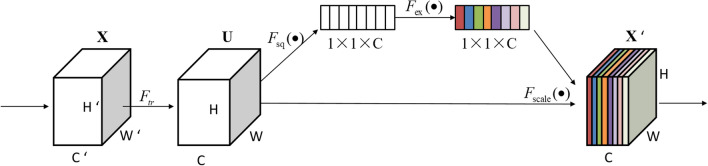
A squeeze-and-excitation module.

The structure of the SE module^29^ in Fig. 2 is shown in Fig. 3.

As shown in Fig. 3, X is mapped to the feature map U through any given transformation, such as convolution. For the feature U, we first make a feature descriptor by a squeezing operation. Then an excitation operation is followed, which uses a simple self-gating mechanism, takes feature descriptors as input, and generates a set of modulation weights for each channel. Finally, these weights are applied to generate the output of the SE module. These outputs can be sent directly to the subsequent layers of the network. By the above operation steps, useful global features can be selectively extracted.

**Figure 4:**
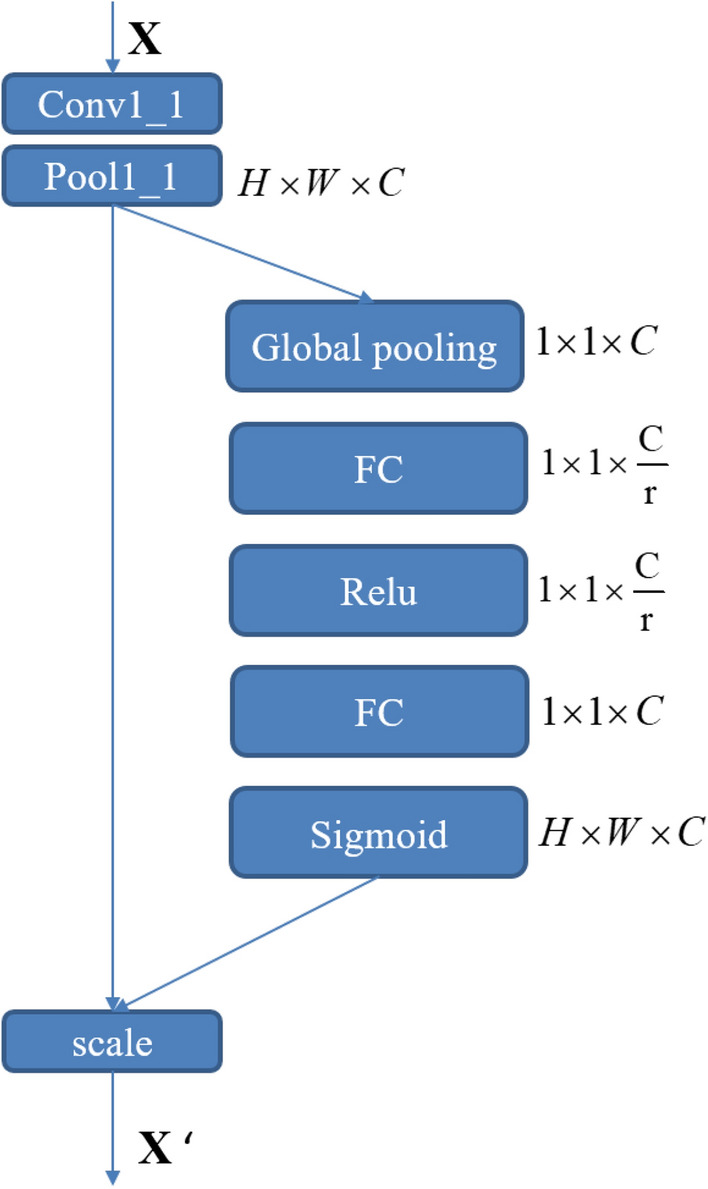
SE block.

The specific structure of the SE block we constructed is shown in Fig. 4.

As shown in Fig. 4, we firstly use global average pooling as a Squeeze operation. Secondly, we use two fully connected layers to form a Bottleneck structure to model the correlation between channels. Thirdly, the sigmoid function is used to obtain the normalized weight between 0 and 1. Finally, a Scale operation is used to weigh the normalized weights to the features of each channel. The *r* in Fig. 4 is the reduction ratio, which is used to reduce the computational cost of the network. We take $r=8$.

### Fractional HS optical flow model

#### Derivation process

Optical flow is a two-dimensional velocity field generated by the movement of the target object. Through the analysis of the two-dimensional velocity field, information such as the speed and direction of the target object’s movement can be obtained. Since the methods of Horn and Schunck (HS)^30^ and Lucas and Kanade (LK)^31^ were proposed, the optical flow algorithm has developed rapidly on this basis. However, the performance of most methods is easily affected by image noise, illumination changes, irregular movement of the target object, etc., and it is difficult to completely extract the detailed features of the target object. Here we have improved the traditional HS algorithm, using fractional calculus to replace the integer calculus in the HS algorithm. The use of fractional order to improve the optical flow algorithm is to ensure the light weight of the network structure and the accuracy of recognition, because using deep learning methods to extract the optical flow map increases the parameters of the network. The quality of the optical flow map extracted using the traditional optical flow algorithm is not good, which affects the recognition accuracy, so we use the fractional order method to improve the traditional method, thus extracting a high quality optical flow map without increasing the parameters of the network. Because fractional calculus has a higher degree of neighborhood pixel correlation and higher calculation accuracy, the extracted target object has more complete details. The model implementation process is as follows.

Assuming that the gray value of the pixel $\left( x,y \right)$ in the image at time *t* is $I\left( x,y,t \right)$. After a very short time $\Delta t$, the gray value becomes $I\left( x+\Delta x,y+\Delta y,t+\Delta t \right)$. Since $\Delta t$ changes in a very short time, it is considered that the following equation holds:

1 $$\begin{aligned} I(x+\Delta x,y+\Delta y,t+\Delta t)=I(x,y,t). . \end{aligned}$$

Using Taylor expansion to expand the left side of formula (1), we get

2 $$\begin{aligned} I(x,y,t)+\frac{\partial I}{\partial x}dx+\frac{\partial I}{\partial y}dy+\frac{\partial I}{\partial t}dt=I(x,y,t). \end{aligned}$$

According to formula (2), the constraint equation of optical flow can be obtained as:

3 $$\begin{aligned} {{I}_{x}}u+{{I}_{y}}v+{{I}_{t}}=0, \end{aligned}$$

where $${{I}_{x}}=\frac{\partial I}{\partial x},{{I}_{y}}=\frac{\partial I}{\partial y},{{I}_{t}}=\frac{\partial I}{\partial t},u=\frac{dx}{dt},v=\frac{dy}{dt}$$.

It can be seen from formula (3) that a constraint equation has two unknown parameters (*u* and *v*), but two unknowns cannot be solved according to one equation, so other constraint equations need to be introduced to solve for these two unknowns. Horn and Schunck^30^ proposed a global smoothing constraint, that is, the changes of *u* and *v* with the movement of pixels are slow, and the changes in the local area are not large, especially when the targets do not deform rigid motion, the space velocity of the local area the rate of change is 0. The global smoothing constraint equation is shown as below:

4 $$\begin{aligned} \zeta _{\text {c}}^{2}=\left( \frac{\partial u}{\partial x}\right) ^{2}+\left( \frac{\partial u}{\partial y}\right) ^{2}+\left( \frac{\partial v}{\partial x}\right) ^{2}+\left( \frac{\partial v}{\partial y}\right) ^{2}. \end{aligned}$$

For all pixels, the sum of the optical flow constraint term and the velocity smoothing constraint term needs to be satisfied as a minimum, so the following minimization equation can be established:

5 $$\begin{aligned} {{\zeta }^{2}}\text {=}\min \int _{\Omega }{({{({{I}_{x}}u+{{I}_{y}}v+{{I}_{t}})}^{2}}+\lambda (\left( \frac{\partial u}{\partial x}\right) ^{2}+\left( \frac{\partial u}{\partial y}\right) ^{2}+{\left( \frac{\partial v}{\partial x}\right) ^{2}}+\left( \frac{\partial v}{\partial y}\right) ^{2}))d\Omega }, \end{aligned}$$

where $\Omega \in R$, $\lambda$ is the coefficient of the smoothing constraint term.

The improved fractional HS optical flow model is shown as below:

6 $$\begin{aligned} \zeta ^{2}\!=\!\min \int _{\Omega }\left( I_{x} u\!+\!I_{y} v\!+\!I_{t}\right) ^{2}\!+\!\lambda \left( \left|D_{x}^{\alpha } \mathrm {u}\right|^{2}\!+\!\left|\mathrm {D}_{y}^{\alpha } \mathrm {u}\right|^{2}\!+\!\left|\mathrm {D}_{x}^{\alpha } \mathrm {v}\right|^{2}\!+\!\left|\mathrm {D}_{y}^{\alpha } \mathrm {v}\right|^{2}\right) d \Omega , \end{aligned}$$

where $\alpha$ is the fractional order. When $\alpha \text {=}1$, formula (6) is same to formula (5).

In order to obtain the Euler–Lagrangian equation corresponding to formula (6), we assume that $u^{*}(x,y)$ and $v^{*}(x,y)$ are expected functions, so that for any test function $\eta (x,y)$ and $\varphi (x,y)\in {{C}^{\infty }}$, *u* and *v* are defined as

7 $$\begin{aligned} u(x, y)=u^{*}(x, y)+\varepsilon \eta (x, y) \\v(x, y)=v^{*}(x, y)+\varepsilon \varphi (x, y), \end{aligned}$$

where $\varepsilon \in R$. Thus, for formula (7), their Riemann–Liouville fractional derivative with respect to *x* and *y* are

8 $$\begin{aligned} D_{x}^{\alpha } u(x, y)=D_{x}^{\alpha } u^{*}(x, y)+\varepsilon D_{x}^{\alpha } \eta (x, y) \\ D_{y}^{\alpha } u(x, y)=D_{y}^{\alpha } u^{*}(x, y)+\varepsilon D_{y}^{\alpha } \eta (x, y) \\ D_{x}^{\alpha } v(x, y)=D_{x}^{\alpha } v^{*}(x, y)+\varepsilon D_{x}^{\alpha } \varphi (x, y) \\ D_{y}^{\alpha } v(x, y)=D_{y}^{\alpha } v^{*}(x, y)+\varepsilon D_{y}^{\alpha } \varphi (x, y). \end{aligned}$$

Substituting formulas (7) and (8) into (6), we can get the following equation:

9 $$\begin{aligned} \zeta ^{2}(\varepsilon )=& \min \int _{\Omega }\left( \left( I_{x}\left( u^{*}+\varepsilon \eta \right) +I_{y}\left( v^{*}+\varepsilon \varphi \right) +I_{t}\right) ^{2}+\lambda \left( \left|D_{x}^{\alpha } u^{*}+\varepsilon D_{x}^{\alpha } \eta \right|^{2}\right. \right. \\& \left. +\left|D_{y}^{\alpha } u^{*}+\varepsilon D_{y}^{\alpha } \eta \right|^{2}+\left|D_{x}^{\alpha } v^{*}+\varepsilon D_{x}^{\alpha } \varphi \right|^{2}+\left|D_{y}^{\alpha } v^{*}+\varepsilon D_{y}^{\alpha } \varphi \right|^{2}\right) d \Omega . \end{aligned}$$

In order to find the extreme value of formula (9), differentiate it and set $\varepsilon =0$, we get

10 $$\begin{aligned} \begin{aligned} {{\zeta }^{2}}'(0)=&\min \int _{\Omega }\left( \left( I_{x} u^{*}+I_{y} v^{*}+I_{t}\right) \left( \eta I_{x}+\varphi I_{y}\right) \right) +\lambda \left( \left( D_{x}^{\alpha } u^{*}\right) D_{x}^{\alpha } \eta \right. \\&\left. +\left( D_{y}^{\alpha } u^{*}\right) D_{y}^{\alpha } \eta +\left( D_{x}^{\alpha } v^{*}\right) D_{x}^{\alpha } \varphi +\left( D_{y}^{\alpha } v^{*}\right) D_{y}^{\alpha } \varphi \right) d \Omega . \end{aligned} \end{aligned}$$

Putting ${{\zeta }^{2}}'(0)=0$ and the coefficients $\eta$ and $\varphi$ are arbitrary values, we get

11 $$\begin{aligned} \left( I_{x} u^{*}+I_{y} v^{*}+I_{t}\right) I_{x}+\lambda \left( D_{x}^{\alpha } * D_{x}^{\alpha } u^{*}+D_{y}^{\alpha *} D_{y}^{\alpha } u^{*}\right) =0 \\ \left( I_{x} u^{*}+I_{y} v^{*}+I_{t}\right) I_{y}+\lambda \left( D_{x}^{\alpha } * D_{x}^{\alpha } v^{*}+D_{y}^{\alpha } * D_{y}^{\alpha } v^{*}\right) =0. \end{aligned}$$

The above derivation results are our improved model for extracting optical flow.

#### Comparison experiment

**Figure 5:**
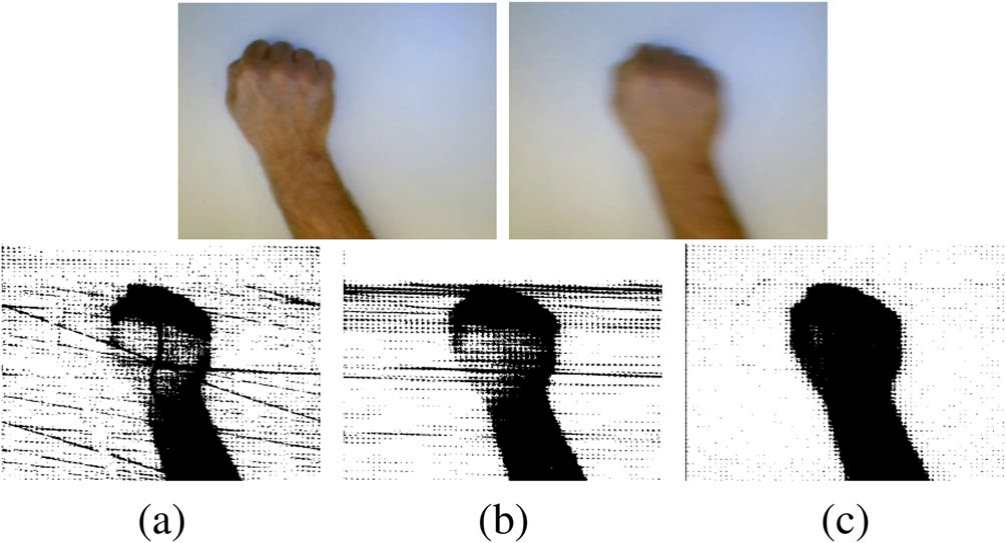
Comparison of optical flow diagrams. (**a**) traditional HS model, (**b**) traditional LK model, (**c**) our model.

To intuitively compare the effects of the optical flow extraction by the improved HS model with that by other models, we randomly select a video in the Northwestern University Hand Gesture dataset^11^, extract two frames of images in the video, and employ the traditional HS model and LK model as the compared models. The comparison result is shown as in Fig. 5.

It can be seen intuitively from Fig. 5 that the details of the optical flow diagram extracted by the HS model and the LK model are poorly processed. The optical flow diagram extracted by our model is relatively complete, and the extraction of details is also relatively complete, which illustrates the effectiveness of our model.

### Key frame extraction

Although video-type data has a strong ability to transmit information, there is too much redundant information. To reduce redundant information and make the transmission of information more efficient, it is necessary to process the video. The extraction of keyframes is one of the important means of processing video data. Because the keyframe extraction is to extract the representative frames in the video, which is consistent with the idea of clustering, the clustering algorithm can be applied to the extraction of keyframes. The algorithm fully considers the connection between frames and can describe the main content of the video specifically.

Traditional clustering algorithms require certain prior knowledge to determine the initial parameters. Most of the initial parameters need to be manually specified, and it is difficult to determine whether the initial parameters are optimal. To obtain effective keyframes, we have improved the traditional clustering algorithm. Because the hierarchical clustering algorithm does not need to specify the optimal initial parameters in advance, we first use the hierarchical clustering algorithm to get the initial clustering results. Then the initial clustering result is used as the input of the traditional clustering algorithm. At this time, the initial parameters of the traditional clustering algorithm can be specified by the initial clustering results.

To facilitate the experiment and reduce the amount of calculation, we use the HSV histogram method to reduce the dimensionality of the image data. First, the RGB color space is mapped to the HSV color space. Then the H component is divided into 12 parts, and the S and V components are divided into 5 equal parts. Finally, the minimum value at the corresponding index of the HSV histogram of the two frames is accumulated, and the value is between 0 and 1. The calculation formulas are as follows.

12 $$\begin{aligned}S_{H}(f, Cont)=\sum _{i=1}^{12} \min \left( H(i), \text{ Cont } _{-} H(i)\right) \\ S_{S}(f, Cont)=\sum _{j=1}^{5} \min \left( S(j), \text{ Cont } _{-} S(j)\right) \\ S_{V}(f, Cont)=\sum _{k=1}^{5} \min \left( V(k), \text{ Cont } _{-} V(k)\right) \\ h=S_{H}+S_{S}+S_{V}, \end{aligned}$$

where *f* is the target frame, *Cont* is the contrast frame, and *h* is the similarity.

The specific process of the improved keyframe extraction algorithm is shown as in Algorithm 1.

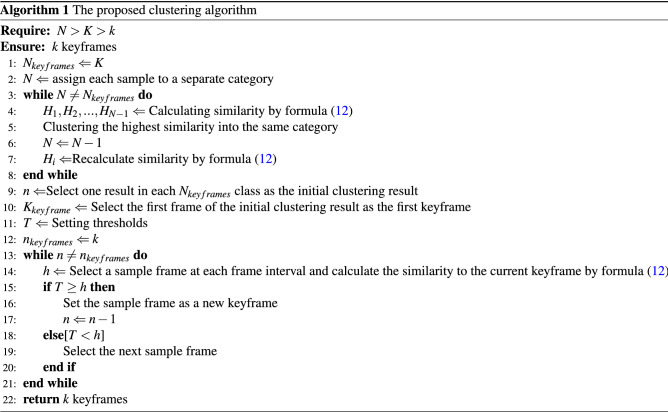



**Figure 6:**
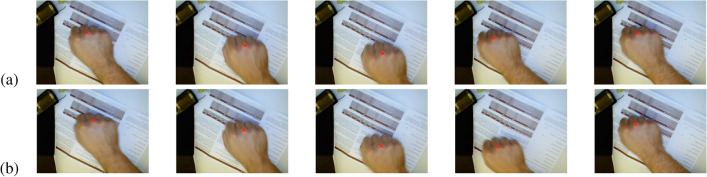
Clockwise circle. (**a**) Traditional clustering algorithms, (**b**) our clustering algorithm.

**Figure 7:**
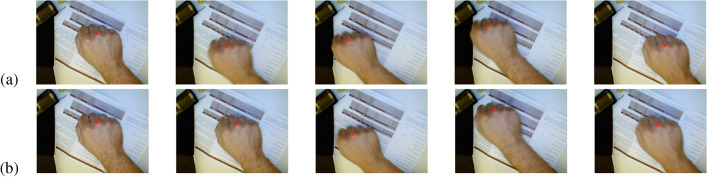
Cross. (**a**) Traditional clustering algorithms, (**b**) our clustering algorithm.

The key frame extraction algorithm is proposed to make the motion trajectory of the extracted key frame sequences closer to the original motion trajectory, thus making it a better alternative to the video datasets for accurate dynamic gesture recognition. To visually compare our clustering algorithm with the traditional k-means clustering algorithm, we have chosen the “clockwise circle” and “cross” types for comparison. The experimental results are shown as in Figs. 6 and 7.

**Figure 8:**
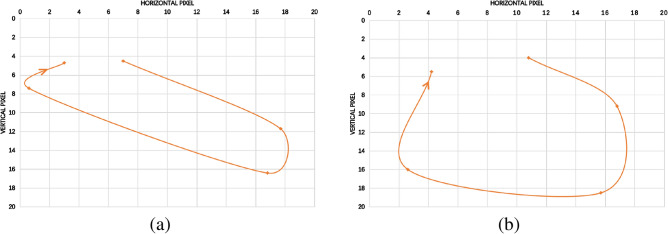
Clockwise circle movement trajectory. (**a**) Traditional clustering algorithms, (**b**) proposed clustering algorithm.

**Figure 9:**
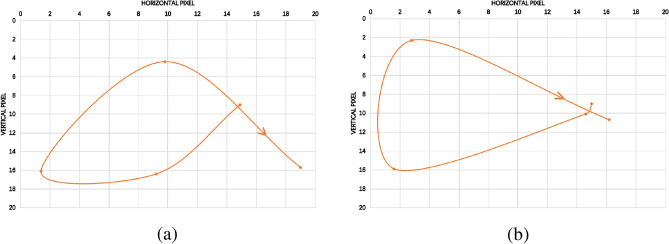
Cross movement trajectory. (**a**) Traditional clustering algorithms, (**b**) proposed clustering algorithm.

To further illustrate the advantages of our algorithm, we select the trajectory of a point on the hand (point “X” in Figs. 6 and 7) in the keyframe sequence to show the effect of the algorithm. The trajectories corresponding to Figs. 6 and 7 are shown as in Figs. 8 and 9, respectively.

As can be seen from Figs. 8 and 9, for both examples, the movement trajectories of the keyframes obtained by our proposed algorithm are easier to recognized.

### Data augmentation

**Figure 10:**
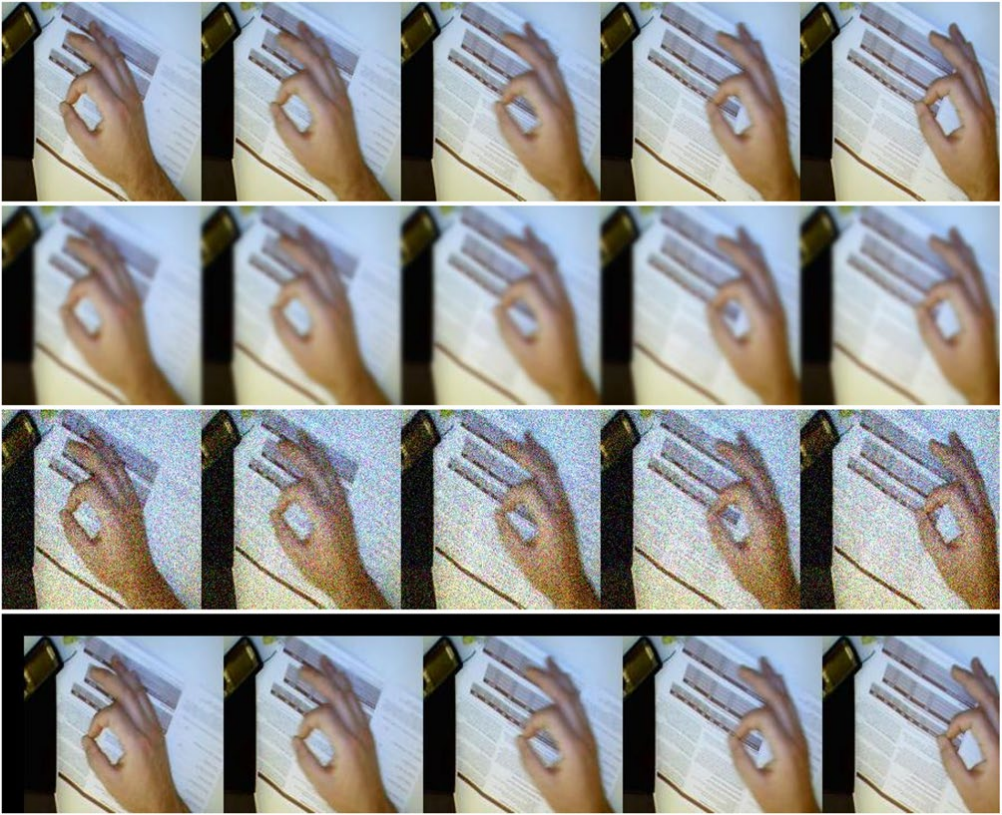
Data enhancement. From top to bottom: original image, blur operation, add noise, translation operation.

In the training process of the network, data enhancement is one of the common methods to prevent overfitting. Commonly used data enhancement methods generally include translation, rotation, flipping, and adding noise. For the proposed dynamic gesture recognition method, the feature map used is a continuous process from left to right, and operations such as rotation and flipping will change the characteristics of the image data. Therefore, we use the operations of shifting pictures, blurring pictures, and adding noise to achieve data enhancement, which expands the data set by 4 times and effectively reduces the possibility of overfitting. The effect of data enhancement is shown as in Fig. 10.

It can be seen from Fig. 10 that the image enhancement technology we used adds three new image data without changing the characteristics of the image information, which improves the diversity of the datasets. Generated frames from the same frame are included in the same subset (training, validation, or testing).

## Experiments and analysis

In this section, we will firstly introduce the two selected public datasets. Secondly, we will explain in detail how to use these two datasets to complete related experiments. Finally, we compare our network with other networks in terms of model parameters, training accuracy, and other aspects to objectively verify the pros and cons of our model.

### Datasets

To verify the effectiveness of the proposed method, we conducted related experiments on two public datasets (Cambridge Hand Gesture datasets^10^ and Northwestern University Hand Gesture datasets^11^).

Cambridge Hand Gesture datasets^10^ contain a total of 9 gesture categories, consisting of 3 gesture shapes (flat, expand, V-shaped) and 3 basic actions (left, right, contract). There are 100 sets of data for each category, and the data is saved in the form of video clips, with a total of 900 video clips.

Northwestern University Hand Gesture datasets^11^ include 10 gesture categories, namely: move right, move left, rotate up, rotate down, move downright, move right-down, clockwise circle, counterclockwise, “Z” and cross. In each category, 15 persons participated in the collection and made 7 gestures (fist, hand, hold, index, side hand, side index, and thumb). There are 105 videos in each category, and there are a total of 1050 videos in the data set.

Both of these two datasets have a certain degree of complexity and can comprehensively verify the pros and cons of the proposed model. 60% of the database is used as the training set, 20% as the validation set, and 20% as the test set. The distribution details of the datasets in the experiment are shown as in Table 1.

**Table 1:** Specific information of the data set used in the experiment.

Dataset	Categories	Videos	Training	Validation	Testing
Cambridge	9	900	540	180	180
Northwestern	10	1050	630	210	210

### Experimental environment

Our experimental environment is: GeForce GTX 1080 Ti GPU, 2.40GHz 6-core CPU, Python 3.6, cuda 10.1, cuDNN 7.6, Tensorflow-GPU 2.3.0.

The specific structure of the proposed network is shown as in Fig. 2. During the training process, the initial learning rate is 0.001, the batch size is 2, and the number of iterations is 500. The optimizer selects Adam and sets the parameter $\beta 1=0.9,\beta 2=0.999$.

For the processing of the datasets, firstly, we use the improved optical flow method to extract optical flow frames from the two datasets. Then we use the proposed clustering algorithm to extract the keyframes of the original frames and the optical flow frames, and the image size of each frame is $640\times 480$. Before the splicing operation, to ensure that the spliced image is not too large, the size of the keyframes is modified to $180\times 180$. After completing the splicing operation, the Cambridge Hand Gesture datasets^10^ obtained 900 original feature maps and 900 optical flow feature maps, and the Northwestern University Hand Gesture datasets^11^ obtained 1050 original feature maps and 1050 optical flow feature maps, respectively. Finally, the data is enhanced by blurring the image, adding noise, and shifting the image and the datasets are enlarged by 4 times. During training, 4320 feature maps in the Cambridge Hand Gesture datasets^10^ are selected as the training set, 1440 feature maps as the validation set and 1440 feature maps as the testing set. We select 5040 feature maps in Northwestern University Hand Gesture datasets^11^ as the training set, 1680 feature maps as the validation set and 1680 feature maps as the testing set.

### Experiment evaluation

**Figure 11:**
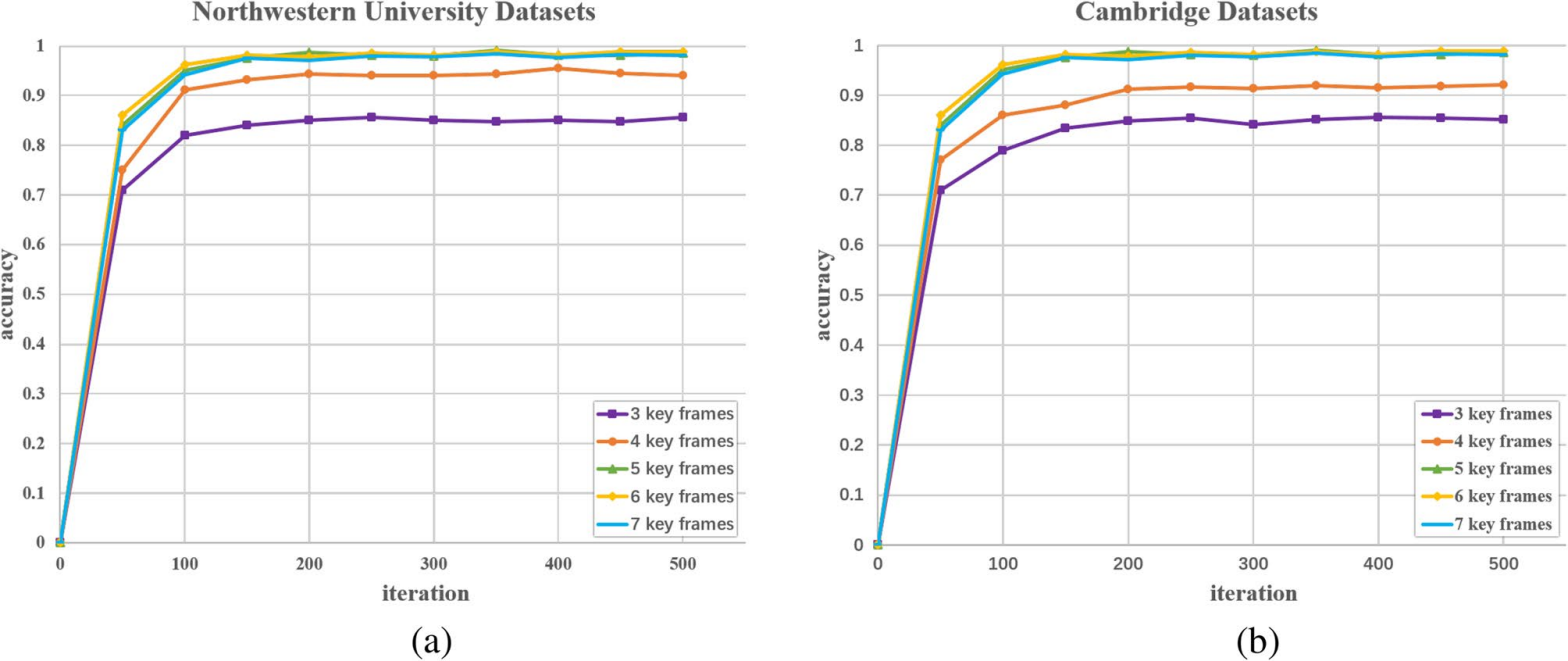
Accuracy curve. (**a**) Northwestern University datasets, (**b**) Cambridge datasets.

When analyzing the network performance, in order to determine how many keyframes work best when extracted for training. We chose to use 3, 4, 5, 6, 7 keyframes for the comparative analysis. The accuracy curves are shown as in Fig. 11.

As can be seen from Fig. 11, when the number of keyframes is 3 and 4, the accuracy obtained will be significantly smaller than the other 3 cases. When the number of keyframes is 6 and 7, the accuracy is not much different from that when the number of keyframes is 5. So after comprehensive consideration, we choose the number of keyframes as 5 for the relevant experiments.

When designing the network structure, in order to determine how many SE blocks can be used for each branch to get better recognition. We chose to use 1, 2, 3, 4, 5 SE blocks respectively for the experiment, and the results are shown in Table 2.

**Table 2:** Accuracy comparison of network structure.

SE block	Top-1 accuracy	
	Northwestern	Cambridge
One	83.87%	84.61%
Two	91.14%	92.49%
Three	97.64%	98.62%
Four	97.81%	**98.74%**
Five	**97.83%**	98.72%

Significant values are in bold.

From Table 2, it can be seen that when each branch is made up of 3 SE blocks, there is a great improvement compared to 1 and 2. However, when each branch is composed of 4 and 5 SE blocks, the improvement in accuracy is not significant and increases the parameters of the network. Therefore, we use the structure of 3 SE blocks per branch.

To intuitively show the effectiveness of our method, we have compared it with some other methods using these two datasets, the comparison results are as shown in Tables 3 and 4.

**Table 3:** Compares our method with state-of-the-art methods on the Northwestern University Hand Gesture datasets.

Northwestern	Methods	Top-1 accuracy
Shen et al.^11^	Motion Divergence fields	95.8%
Liu et al.^32^	Genetic programming	96.1%
Tang et al.^33^	Key frames + Feature fusion	96.9%
Ours	Key frames splicing + feature fusion	**97.6%**

Significant values are in bold.

**Table 4:** Compares our method with state-of-the-art methods on the Cambridge datasets.

Cambridge	Methods	Top-1 accuracy
Kim et al.^10^	Tensor canonical correlation analysis	82.4%
Liu et al.^32^	Genetic programming	85.5%
Lui et al.^34^	Tangent bundle	91.3%
Wong et al.^35^	Probabilistic latent semantic analysis	91.4%
Baraldi et al.^36^	Dense trajectories + hand segmentation	94.1%
Zhao et al.^37^	Information theoretic	96.2%
Tang et al.^33^	Key frames + feature fusion	98.2%
Ours	Key frames splicing + feature fusion	**98.6%**

Significant values are in bold.

It can be seen from Tables 3 and 4 that the accuracy of the proposed method is 97.6% on the Northwestern University datasets and 98.6% on the Cambridge datasets, both of which are better than other methods.

Furthermore, we compare the proposed model with the common used 3D model for dynamic gesture recognition in term of accuracy, parameters, and FLOPs. The comparison results on the Northwestern University Hand Gesture datasets are shown as in Table 5.

**Table 5:** The performance of gesture recognition on the Northwestern University datasets.

Model	Top-1 accuracy	Params (M)	FLOPs (G)
C3D^8^	89.36%	63.74	38.59
P3D^38^	97.62%	24.98	8.15
I3D^39^	**98.88%**	12.36	27.82
Ours	97.64%	**0.44**	**4.22**

Significant values are in bold.

It can be obtained a conclusion from the results in Table 5 that our model has the smallest parameters and FLOPs while ensuring high accuracy. It shows that the recognition efficiency of our method is more efficient.

To compare the efficiency of the various algorithms more intuitively, we calculated the time taken by the model to classify a test sequence. The results are shown in Table 6. The time of our algorithm is 9.93 s on the Northwestern University gesture dataset and 4.02 s on the Cambridge gesture dataset, both of which are more significant improvements over previous algorithms. When conducting experiments we found that the feature extraction process and the size of the feature map have a large impact on the time required. Therefore, the keyframe extraction algorithm and the fusion rules of the feature maps can be given priority in the subsequent improvements.

**Table 6:** Computation time for classifying a test sequence.

Method	Time	
	Northwestern	Cambridge
Zhao et al.^37^	11.78 s	5.34 s
Liu et al.^32^	13.32 s	6.45 s
Tang et al.^33^	10.89 s	4.31 s
Ours	9.93 s	4.02 s

To intuitively test the accuracy of each category recognition, we made a new 100 keyframe mosaics for each category of the Northwestern University data set and Cambridge Hand Gesture data set to make predictions. The confusion matrix of the prediction results is as Tables 7 and 8, where we note the ten categories of move right, move left, rotate up, rotate down, move down-right, move right-down, clockwise circle, counterclockwise circle, “Z” and cross with A, B, C, D, E, F, G, H, I, and J, respectively. Similarly, we denote the Cambridge gesture datasets “‘ flat and leftward ”, “flat and rightward”, “flat and contract”, “spread and leftward”, “spread and rightward”, “spread and contract”, “V-shape and leftward”, “V-shape and rightward” and “ V-shape and contract ” these nine categories are denoted by A, B, C, D, E, F, G, H, and I, respectively.

**Table 7:** Identification confusion matrix of the Northwestern University hand gesture dataset.

	A	B	C	D	E	F	G	H	I	J
A	**96**	0	1	2	1	0	0	0	0	0
B	1	**96**	3	0	0	0	0	0	0	0
C	4	1	**95**	0	0	0	0	0	0	0
D	2	0	0	**93**	5	0	0	0	0	0
E	1	1	0	7	**91**	0	0	0	0	0
F	1	0	0	2	0	**94**	3	0	0	0
G	0	0	0	1	0	3	**95**	1	0	0
H	2	0	0	0	0	1	2	**95**	0	0
I	0	0	0	1	2	0	2	0	**95**	0
J	0	0	0	2	0	0	0	0	0	**98**

Significant values are in bold.

**Table 8:** Identification confusion matrix of the Cambridge hand gesture dataset.

	A	B	C	D	E	F	G	H	I
A	**97**	0	1	1	0	0	1	0	0
B	1	**96**	0	0	2	0	0	1	0
C	0	0	**96**	0	0	2	0	1	1
D	3	1	0	**92**	0	0	3	0	1
E	0	2	0	0	**96**	0	0	2	0
F	1	0	2	0	0	**94**	0	0	3
G	2	1	1	1	0	0	**95**	0	0
H	0	0	0	0	4	1	2	**93**	0
I	0	0	1	0	0	4	0	0	**95**

Significant values are in bold.

**Figure 12:**
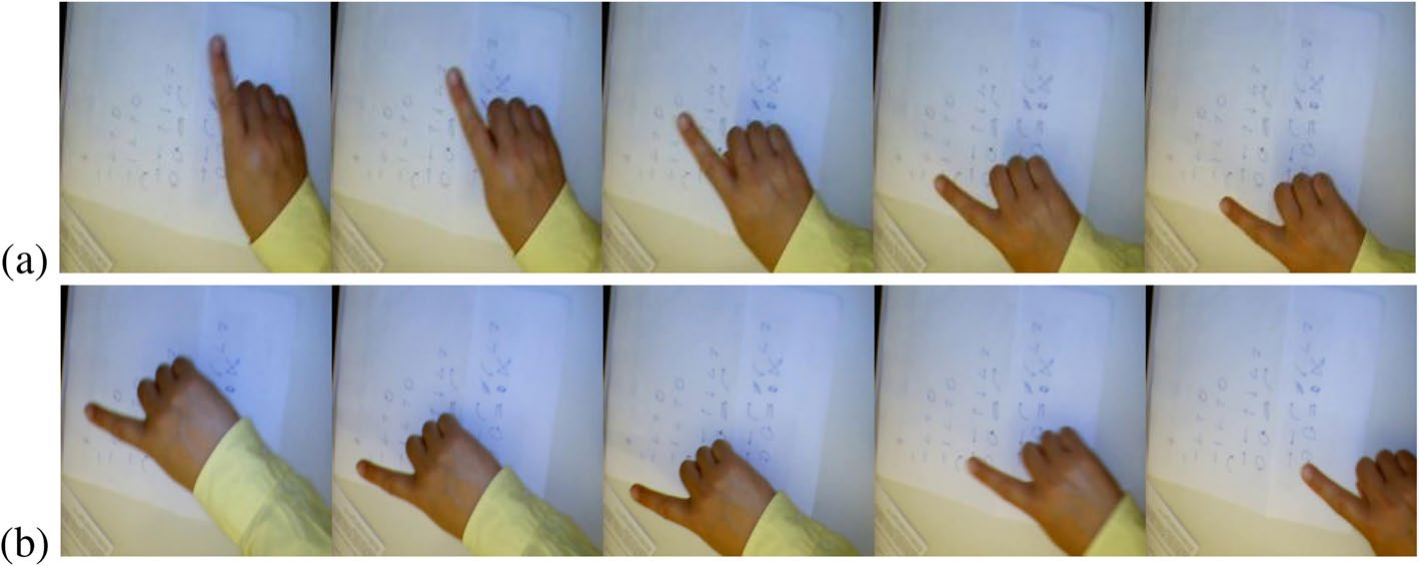
The sample frames of the confused gestures. (**a**) rotate down, (**b**) move downright.

It can be seen from the recognition confusion matrix that most categories can achieve accurate prediction, but D and E (“rotate down” and “move down-right”) in Table 7 are easy to be confused. We compare the feature maps of these two categories and found that when represented by a keyframe mosaic map, the movement trajectories of the two categories are similar, which is more likely to cause misrecognition. The comparison of the feature maps of these two categories is shown as in Fig. 12.

For improving this problem to get a better performance, to add feature maps of these two types of actions may be a good method to make the network can learn more detailed features.

### Ablation study

To verify the effectiveness of the fractional-order HS optical flow algorithm and the key frame extraction algorithm we used, we conducted ablation experiments to analyze the effect of the optical flow algorithm and the key frame extraction algorithm on the recognition accuracy. As shown in Tables 9 and 10.

**Table 9:** Accuracy comparison of optical flow algorithm.

Method	Top-1 accuracy	
	Northwestern	Cambridge
HS algorithm	94.59%	95.03%
LK algorithm	96.11%	96.83%
Our algorithm	**97.64%**	**98.62%**

Significant values are in bold.

**Table 10:** Accuracy comparison of keyframe algorithm.

Method	Top-1 accuracy	
	Northwestern	Cambridge
K-means algorithm	93.44%	94.81%
Our algorithm	**97.64%**	**98.62%**

Significant values are in bold.

To illustrate the effectiveness of the proposed spatial feature and temporal feature fusion strategy, we conduct ablation experiments to analyze the impact of the original keyframes and optical flow keyframes on the recognition accuracy, and the results are shown as in Table 11.

**Table 11:** Accuracy comparison of ablation experiments.

Method	SE module	Top-1 accuracy	
		Northwestern	Cambridge
Original frame + Optical flow frame	$\checkmark$	**97.64%**	**98.62%**
Original frame + Optical flow frame		96.52%	97.23%
Original frame	$\checkmark$	83.67%	88.47%
Original frame		81.44%	87.18%
Optical flow frame	$\checkmark$	87.21%	79.13%
Optical flow frame		85.79%	78.07%

Significant values are in bold.

It can be seen from Table 11 that the proposed fusion strategy of the original keyframe and the optical flow keyframe can get over 10% than the training alone in accuracy. Indicating that the proposed fusion strategy can effectively improve the recognition accuracy.

To enable the network to selectively extract useful global features, we added the SE module to the network model. It can also be concluded from the results shown as in Table 11 that the final accuracy of the recognition network with the SE module is approximately 1.5% higher than that of the recognition network without the SE module.

## Conclusion

For the problems of high network complexity, high computational difficulty, and slow training speed in the current dynamic gesture recognition field, we propose a dynamic gesture recognition method based on feature fusion and a 2D convolutional neural network. We use the fractional-order model to extract the optical flow frames of the video, and creatively incorporate the fractional-order into the neural network. Then extract the keyframes of the original frame and the optical flow frame, and replace the video with the keyframe mosaic, which greatly reduces the redundant information in the video data. With experimental verification results, the accuracy of the proposed method is 97.6% on the Northwestern University datasets and 98.6% on the Cambridge datasets, which surpasses other methods using the two datasets. In terms of network parameters, our network parameters are only 0.44 M, which is tens of times smaller compared to the commonly used 3D CNN model, and also the FLOPs are very much smaller. To further demonstrate the efficiency of our proposed algorithm, we compare the computation time for classifying a test sequence. The results show that our proposed algorithm has some improvement in the time required for recognition under the condition of the highest accuracy. To show the effectiveness of the proposed spatial feature and temporal feature fusion strategy, we conduct an ablation experiment to compare the accuracy of recognition with only spatial features and only temporal features. The results show that the accuracy of the proposed fusion strategy is higher than that of only spatial and temporal features on the Northwestern University datasets and the Cambridge datasets.

## References

[1] Rautaray SS, Agrawal A (2012). Vision based hand gesture recognition for human computer interaction: A survey. Artif. Intell. Rev..

[2] Wang C, Liu Z, Chan SC (2015). Superpixel-based hand gesture recognition with kinect depth camera. IEEE Trans. Multimed..

[3] Lv Z, Halawani A, Feng S, ur Réhman S, Li H (2015). Touch-less interactive augmented reality game on vision-based wearable device. Pers. Ubiquit. Comput..

[4] Ren, Z., Yuan, J. & Zhang, Z. Robust hand gesture recognition based on finger-earth mover’s distance with a commodity depth camera. In *Proceedings of the 19th ACM international conference on Multimedia* (2011).

[5] Luzanin O, Plancak M (2014). Hand gesture recognition using low-budget data glove and cluster-trained probabilistic neural network. Assem. Autom..

[6] Zhuang H-W, Yang M, Cui Z-X, Zheng Q (2017). A method for static hand gesture recognition based on non-negative matrix factorization and compressive sensing. IAENG Int. J. Comput. Sci..

[7] Zheng Q, Tian X, Liu S, Yang M, Wang H (2018). Static hand gesture recognition based on gaussian mixture model and partial differential equation. IAENG Int. J. Comput. Sci..

[8] Tran, D., Bourdev, L. D., Fergus, R., Torresani, L. & Paluri, M. Learning spatiotemporal features with 3d convolutional networks. In *2015 IEEE International Conference on Computer Vision (ICCV)* 4489–4497 (2015).

[9] Chen D, Sheng H, Chen Y, Xue D (2013). Fractional-order variational optical flow model for motion estimation. Philos. Trans. R. Soc. A Math. Phys. Eng. Sci..

[10] Kim, T.-K., Wong, S.-F. & Cipolla, R. Tensor canonical correlation analysis for action classification. In *2007 IEEE Conference on Computer Vision and Pattern Recognition* 1–8 (2007).

[11] Shen X, Hua G, Williams L, Wu Y (2012). Dynamic hand gesture recognition: An exemplar-based approach from motion divergence fields. Image Vis. Comput..

[12] Wang X, Xia M, Cai H, Gao Y, Cattani C (2012). Hidden-Markov-models-based dynamic hand gesture recognition. Math. Probl. Eng..

[13] Oreifej, O. & Liu, Z. Hon4d: Histogram of oriented 4d normals for activity recognition from depth sequences. In *2013 IEEE Conference on Computer Vision and Pattern Recognition* 716–723 (2013).

[14] Chen F-S, Fu C-M, Huang C-L (2003). Hand gesture recognition using a real-time tracking method and hidden Markov models. Image Vis. Comput..

[15] Rahman MH, Afrin J (2013). Hand gesture recognition using multiclass support vector machine. Int. J. Comput. Appl..

[16] Cheng Y (2021). Gesture recognition based on surface electromyography-feature image. Concurr. Comput. Pract. Exp..

[17] Liao S (2021). Occlusion gesture recognition based on improved SSD. Concurr. Comput. Pract. Exp..

[18] Li C-C, Li G, Jiang G, Chen D, Liu H (2020). Surface EMG data aggregation processing for intelligent prosthetic action recognition. Neural Comput. Appl..

[19] Huang L, Fu Q, He M, Jiang D, Hao Z (2021). Detection algorithm of safety helmet wearing based on deep learning. Concurr. Comput. Pract. Exp..

[20] Huang L (2020). Jointly network image processing: Multi-task image semantic segmentation of indoor scene based on cnn. IET Image Process..

[21] Yang Z (2021). Dynamic gesture recognition using surface EMG signals based on multi-stream residual network. Front. Bioeng. Biotechnol..

[22] Weng Y (2021). Enhancement of real-time grasp detection by cascaded deep convolutional neural networks. Concurr. Comput. Pract. Exp..

[23] Duan H (2021). Gesture recognition based on multi-modal feature weight. Concurr. Comput. Pract. Exp..

[24] Liu Z, Hu H, Zhang J (2019). Spatiotemporal fusion networks for video action recognition. Neural Process. Lett..

[25] Karpathy, A. *et al.* Large-scale video classification with convolutional neural networks. In *2014 IEEE Conference on Computer Vision and Pattern Recognition* 1725–1732 (2014).

[26] Simonyan, K. & Zisserman, A. Two-stream convolutional networks for action recognition in videos. In *NIPS* (2014).

[27] Wang, L. *et al.* Temporal segment networks: Towards good practices for deep action recognition. ArXiv abs/1608.00859 (2016).

[28] Molchanov, P., Gupta, S., Kim, K. & Kautz, J. Hand gesture recognition with 3d convolutional neural networks. In *2015 IEEE Conference on Computer Vision and Pattern Recognition Workshops (CVPRW)* 1–7 (2015).

[29] Hu J, Shen L, Albanie S, Sun G, Wu E (2020). Squeeze-and-excitation networks. IEEE Trans. Pattern Anal. Mach. Intell..

[30] Horn BKP, Schunck BG (1981). Determining optical flow. Artif. Intell..

[31] Lucas, B. D. & Kanade, T. An iterative image registration technique with an application to stereo vision. In *IJCAI* (1981).

[32] Liu, L. & Shao, L. Synthesis of spatio-temporal descriptors for dynamic hand gesture recognition using genetic programming. In *2013 10th IEEE International Conference and Workshops on Automatic Face and Gesture Recognition (FG)* 1–7 (2013).

[33] Tang H, Liu H, Xiao W, Sebe N (2019). Fast and robust dynamic hand gesture recognition via key frames extraction and feature fusion. Neurocomputing.

[34] Lui YM, Beveridge JR (2011). Tangent bundle for human action recognition. Face Gesture.

[35] Wong, S.-F., Kim, T.-K. & Cipolla, R. Learning motion categories using both semantic and structural information. In *2007 IEEE Conference on Computer Vision and Pattern Recognition* 1–6 (2007).

[36] Baraldi, L., Paci, F., Serra, G., Benini, L. & Cucchiara, R. Gesture recognition in ego-centric videos using dense trajectories and hand segmentation. In *2014 IEEE Conference on Computer Vision and Pattern Recognition Workshops* 702–707 (2014).

[37] Zhao, Z. & Elgammal, A. Information theoretic key frame selection for action recognition. In *BMVC* (2008).

[38] Qiu, Z., Yao, T. & Mei, T. Learning spatio-temporal representation with pseudo-3d residual networks. In *2017 IEEE International Conference on Computer Vision (ICCV)* 5534–5542 (2017).

[39] Wang, L., Koniusz, P. & Huynh, D. Q. Hallucinating IDT descriptors and i3d optical flow features for action recognition with CNNS. In *2019 IEEE/CVF International Conference on Computer Vision (ICCV)* 8697–8707 (2019).

